# Case report: Filarial infection of a parti-coloured bat: *Litomosa* sp. adult worms in abdominal cavity and microfilariae in bat semen

**DOI:** 10.3389/fvets.2023.1284025

**Published:** 2023-09-21

**Authors:** Jiri Pikula, Vladimir Piacek, Hana Bandouchova, Marie Bartlova, Sarka Bednarikova, Romana Burianova, Ondrej Danek, Petr Jedlicka, Sarka Masova, Monika Nemcova, Veronika Seidlova, Katerina Zukalova, Jan Zukal

**Affiliations:** ^1^Department of Ecology and Diseases of Zoo Animals, Game, Fish and Bees, University of Veterinary Sciences Brno, Brno, Czechia; ^2^Department of Plant Origin Food Sciences, University of Veterinary Sciences Brno, Brno, Czechia; ^3^Department of Pathology and Parasitology, University of Veterinary Sciences Brno, Brno, Czechia; ^4^Biology Centre, Institute of Parasitology, Czech Academy of Sciences, České Budějovice, Czechia; ^5^Institute of Scientific Instruments of the Czech Academy of Sciences v.v.i., Brno, Czechia; ^6^Department of Botany and Zoology, Faculty of Science, Masaryk University, Brno, Czechia; ^7^Institute of Vertebrate Biology, Czech Academy of Sciences, Brno, Czechia

**Keywords:** Chiroptera, *Vespertilio murinus*, electroejaculation, semen quality parameters, semen-borne pathogens, filariasis, *Wolbachia*

## Abstract

**Background:**

Filarial infections have been understudied in bats. Likewise, little is known about pathogens associated with the reproductive system in chiropterans. While semen quality is critical for reproductive success, semen-borne pathogens may contribute to reproductive failure.

**Methods:**

For the first time we performed electroejaculation and used computer-assisted semen analysis to provide baseline data on semen quality in a parti-coloured bat (*Vespertilio murinus*).

**Results:**

The semen quality values measured in the *V. murinus* male appeared high (semen concentration = 305.4 × 10^6^/mL; progressive and motile sperm = 46.58 and 60.27%, respectively). As an incidental finding, however, microfilariae were observed in the bat semen examined. At necropsy, eight adult filarial worms, later genetically identified as *Litomosa* sp., were found in the peritoneal cavity, close to the stomach, of the same particoloured bat male dying as a result of dysmicrobia and haemorrhagic gastroenteritis in a wildlife rescue centre. Histopathology revealed microfilariae in the testicular connective tissue and the epidydimal connective and fat tissues. A PCR assay targeting cytochrome c oxidase subunit 1 confirmed that adult worms from the peritoneal cavity and testicular microfilariae were of the same filarial species. Mildly engorged argasid mite larvae attached to the bat skin proved negative for filarial DNA and the adult filarial worms proved negative for endosymbiont *Wolbachia.*

**Conclusion:**

While the standard filarial life cycle pattern involves a vertebrate definitive host and an invertebrate vector, represented by a blood-sucking ectoparasite, our finding suggests that microfilariae of this nematode species may also be semen-borne, with transmission intensity promoted by the polygynous mating system of vespertilionid bats in which an infected male mates with many females during the autumn swarming. Presence of microfilariae may be expected to decrease semen quality and transmission *via* this route may challenge the success of reproductive events in females after mating. Further investigation will be necessary to better understand the bat-parasite interaction and the life cycle of this filarial worm.

## 1. Introduction

According to a conservative estimate, between 4,000 and 5,000 endoparasite species are believed to infect bats around the world (1). However, while hundreds of nematodes have been described in bats (2), their biology, life cycles and host–parasite specificity and interactions remain largely unknown (3). Onchocercid filarial nematodes, and especially the genera *Litomosa* and *Litomosoides*, occur relatively frequently in both Old World and Nearctic and Neotropical bats of the families Hipposideridae, Miniopteridae, Molossidae, Phyllostomidae, Pteropidae, Rhinolophidae and Vespertilionidae (4–13). Adult *Litomosa* and *Litomosoides* generally reside within the pleural and/or peritoneal cavities of bats; however, cerebral ventricles (14) or the pulmonary artery, the right ventricle of the heart and the portal vein (9), may also be favoured sites for some adult onchocercid filariae.

Aside from *Loa loa* (15), most filarial nematodes of medical and veterinary concern (16) also host the intracellular bacterial symbiont *Wolbachia*. Providing metabolites that filarial nematodes are incapable of synthesising, this endosymbiont is fundamental for key biochemical pathways required for growth and development of larvae and embryogenesis in female worms (17). *Wolbachia* also contributes to the immunopathology of filarial infections (18, 19).

To date, three onchocercid filarial nematodes have been recorded in parti-coloured bats, with *Litomosa ottavianii* (20) and *Litomosa vaucheri* (10) described in the parti-coloured bat (*Vespertilio murinus*) and similar *Litomosa* sp. described in its sibling species, the Asian parti-coloured bat (*Vespertilio sinensis*) (9). The distribution of *V. murinus* ranges over much of Europe to west Asia, Mongolia, northern China and the Russian Far East, where it overlaps with *V. sinensis* (21). Mainly found in cities in the Czech Republic, individuals with injuries are commonly found in high buildings and brought to rescue centres (22).

In October 2021, a *V. murinus* male was brought for examination and treatment to the wildlife rescue centre at the University of Veterinary Sciences in Brno, Czech Republic, after it was found in a block of flats unable to fly. As the animal was otherwise in good condition, we used the opportunity of general anaesthesia to collect a semen sample by electroejaculation. While evaluating the quality of semen collected using computer-assisted semen analysis, we recorded microfilariae as an incidental finding. Adult filarial worms were also later found in the peritoneal cavity of the same male, with histopathology revealing microfilariae in the testes. We then attempted to identify the filarial parasite genetically and test it for presence of the bacterial symbiont *Wolbachia*.

## 2. Materials and methods

### 2.1. Ethics statement

Experimental procedures within the project “Sperm and European bat mating systems” were approved by the Ethical Committee of the University of Veterinary Sciences Brno (document no. 9-2021) and the Ministry of the Environment of the Czech Republic (document no. MZP/2021/630/2084). Semen sample collection was based on a permit issued by the Agency for Nature Conservation and Landscape Protection of the Czech Republic (SR/0249/JM/2021-3). All team members were authorised to handle wild bats according to Czech Certificate of Competency (No. CZ01341; §17, Act No. 246/1992 Coll.).

### 2.2. Semen collection and examination

The male *V. murinus* treated at the wildlife rescue centre at the University of Veterinary Sciences in Brno had a left humerus fractured in diaphysis. The fracture site was open and infected and the distal part of the affected wing was necrotic due to disrupted blood supply; consequently, amputation was employed to save the bat’s life. When animals have no chance of survival in the wild or a poor quality of life in captivity may be expected, euthanasia is considered an appropriate action. In this case, wing amputation leading to disability and permanent captivity of the injured bat was justified by keeping the animal for educational purposes. The surgery was performed under inhalation anaesthesia using isoflurane (Isoflurin 1,000 mg/g; Vetpharma Animal Health, S.L., Spain) delivered through a small mask from a Matrix Technologies VIP 3000 Veterinary Isoflurane Vaporiser (Midmark, United States) using an open non-rebreathing system. The bat was induced and maintained with 5 and 2% isoflurane, respectively, carried with 1.0 L/min oxygen.

As the animal was otherwise in good condition, we used the opportunity of general anaesthesia to collect a semen sample by electroejaculation, using an impulse electroejaculation generator (designed and developed by our team for semen collection from small mammals) connected to a 1 mm diameter rectal probe with electrodes 4 mm from the probe’s tip. Lubricated with ultrasound gel, the probe was inserted approximately 9 mm into the bat’s rectum and positioned against the prostate region. Electrical stimulation was then initiated with intensity ranging from 0.20 to 4 mA (50 Hz), as described elsewhere (23). A series of stimulations was alternated with periods of rest until erection was reached and a drop of semen was seen at the tip of the bat’s penis. The ejaculate was then collected using a laboratory pipette and extended (dilution 1:10 v/v) immediately with a 37°C pre-warmed DMEM-F12 solution (Dulbecco’s Modified Eagle Medium; Biosera, France).

Fresh bat semen characteristics were determined immediately after collection using a Sperm Class Analyser-Computer Assisted Sperm Analysis (SCA-CASA) system (Microptic s.l., Spain), with concentration and motility determination modules and a Nikon eclipse E200LED MV R camera equipped with a Nikon 10 × 0.25 Ph 1 BM WD 7.0 lens (Nikon Corporation, Japan) and an acA 1,300-200uc Basler c-Mount camera (Basler a.g., Germany). Semen concentration (×10^6^/ml) and total motility parameters were measured in a pre-warmed 8 × 2 μL Leja SC 20-01-08-B-CE counting chamber (Cryo Tech s.r.o., Czech Republic).

### 2.3. Pathological examination

The bat’s recovery from general anaesthesia and surgery was uneventful. The animal was housed in a bat box with soft mesh on the inner walls and cloth layers to provide roosting and hiding places along with a water dish for drinking. The bat accepted hand-offered mealworms twice daily from the day following surgery. After 3 weeks, however, the bat stopped feeding and died suddenly.

On necropsy, both the abdominal and thoracic cavities were visually inspected for gross lesions and presence of parasites. We also collected samples for histopathology (lungs, liver, kidney, testicle) and placed these in 10% buffered formalin. Paraffin embedded samples were then sectioned into 5 μm slices on a rotary microtome (RM2255, Leica Microsystems GmbH, Wetzlar, Germany), mounted onto a slide and stained using hematoxylin–eosin (Sigma-Aldrich, United States) as described earlier (24). The bat’s skin was also examined for ectoparasites.

### 2.4. Identification of intestinal microbial agents

During necropsy, the gut contents were collected for DNA isolation with a commercially available E.Z.N.A.® Soil DNA Kit (Omega, United States), following the manufacturer’s instructions. The isolated DNA sample was stored at −20°C, after which a 16S Barcoding Kit (Oxford Nanopore Ltd., United Kingdom) was used to amplify the entire 16S rRNA gene (25 cycles with primers) and prepare amplicons for sequencing. Barcoding was performed using a Bio-Rad Mini cycler (Bio-Rad, United States) and the amplicons purified in line with the manufacturer’s instructions using AMPure XP Beads (Beckmann Coulter, United States). Sequencing was performed with a MinION nanopore sequencer using the reagents supplied with the 16S Barcoding Kit. Reads were filtered and those with a Phred score < 7 were removed from the analysis. A previously described pipeline was used for bioinformatic analysis (25), with individual reads taxonomically assigned using the RDP16S_v.18 database (26). Raw 16S sequences were deposited in the NCBI Sequence Read Archive (SRR25944610).

### 2.5. Molecular identification of the filarial parasite

DNA was extracted from adult worms, from the bat’s testicles and from blood-sucking ectoparasites attached to the skin using the NucleoSpin® Microbial DNA extraction kit (Macherey-Nagel, Germany), following the methodology recommended in the manufacturer’s instructions. The eluted DNA was then stored at −20°C until polymerase chain reaction (PCR) screening.

To identify parasitic filarial species in the samples, a PCR assay targeting the cytochrome c oxidase subunit 1 (COI) was performed, using the universal primers COIintF (5′-TGATTGGTGGTTTTGGTAA-3′) and COIintR (5′-ATAAGTACGAGTATCAATATC-3′) (27). The PCR mixture comprised 10 μL of Phusion Green Hot Start II High-Fidelity PCR Master Mix (Thermo Fischer Scientific, United States), 0.5 μM of each primer and 2 μL of DNA, prepared to a final volume of 20 μL. Amplification was then performed using a TProfessional TRIO Thermocycler (Biometra, Germany) over a total of 40 cycles.

Amplification products were visualised on 2% agarose gel stained with Midori Green Advance DNA Stain (Nippon Genetics Europe, Germany). All samples yielding an amplicon of the appropriate size (689 bp) were purified using the Gel PCR DNA Fragments Extraction Kit (Geneaid, Taiwan). The purified PCR products were commercially sequenced (Macrogen Europe, The Netherlands) and the sequences obtained aligned in Geneious Prime software (Biomatters, New Zealand) and compared with available sequences in GenBank.

Sequences with mixed chromatograms were cloned using the Zero Blunt™ TOPO™ PCR Cloning Kit (Thermo Fisher Scientific, United States). The plasmid DNA acquired was purified from the bacterial culture using the GenElute™ Plasmid Miniprep Kit (Sigma-Aldrich, United States) and sequenced using universal T7/SP6 primers.

### 2.6. *Wolbachia* screening

Two adult filarial worms from the bat’s peritoneal cavity were used for DNA isolation and testing for the presence of the bacterial symbiont *Wolbachia*. We used the PCR protocol for *Wolbachia* detection described previously (28). The method is based on amplification of the 16S rRNA gene fragment (~1,100 bp) using the *Wolbachia*-specific primers: 16S 281F 5′- CTATAGCTGATCTGAGAGGAT-3′ and 16S 1372R 5′-YGCTTCGAGTGAAACCAATTC-3′. Reactions were performed in a 25 μL reaction mixture containing 12.5 μL of Super-Hot Master Mix 2x (Bioron GmbH, Germany), 0.5 μM of each primer and 1 μL of isolated DNA. PCR procedures included a first step of 94°C for 3 min, followed by 35 cycles of 94°C for 45 s, 55°C for 60 s, 72°C for 90 s and a final step of 72°C for 10 min. The amplified DNA products (4 μL) were stained with SERVA DNA Stain G (SERVA Electrophoresis GmbH, Germany) and visualised by electrophoresis on 1.5% agarose gel.

## 3. Results

### 3.1. Bat semen quality

**Figure 1:**
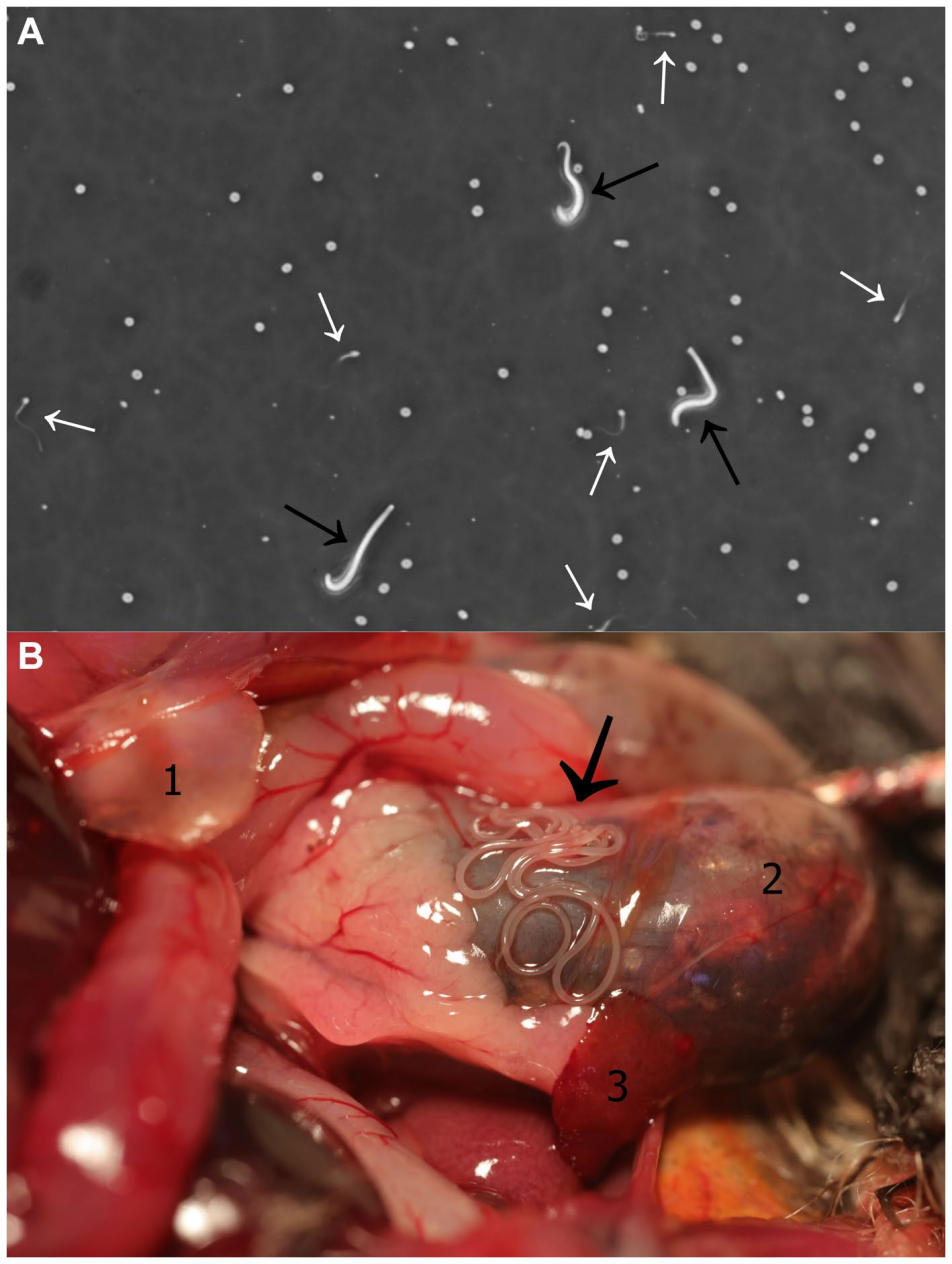
*Litomosa* sp. filarial infection. **(A)** Semen-borne microfilariae in a male parti-coloured bat (*Vespertilio murinus*). Image captured from video footage obtained during a Sperm Class Analyser - Computer Assisted Sperm Analysis. Microfilariae and spermatozoa are indicated with black and white arrows, respectively. **(B)** Filarial nematodes within the abdominal cavity of a male parti-coloured bat. Thread-like adult worms located along the stomach (black arrow). Anatomical structures seen in the figure: (1) *Cartilago xiphoidea*, (2) stomach, (3) spleen.

The semen sample, which was collected within a minute of electrical stimulation, had a total volume of 1 μL and was highly viscous. SCA-CASA revealed the following parameter values: semen concentration = 305.4 × 10^6^/mL; progressive and motile sperm = 46.58 and 60.27%, respectively; rapid velocity = 45.21%; velocity and progressivity: rapid progressive = 24.66%, medium progressive = 21.92%, non-progressive = 13.70%, immotile = 39.73%; average sperm head area = 30.84 μm^2^; speed: curve speed = 141.71 μm/s, average value = 136.90 μm/s, linear speed = 111.71 μm/s, straightness index = 71.94%, linearity index = 67.54%, oscillation index = 87.12%; beat frequency = 5.12 Hz; mucous penetration = 38.64%. In addition to spermatozoa, active microfilariae were also observed in the semen sample (Figure 1A; Supplementary Video S1).

### 3.2. Pathological examination

**Figure 2:**
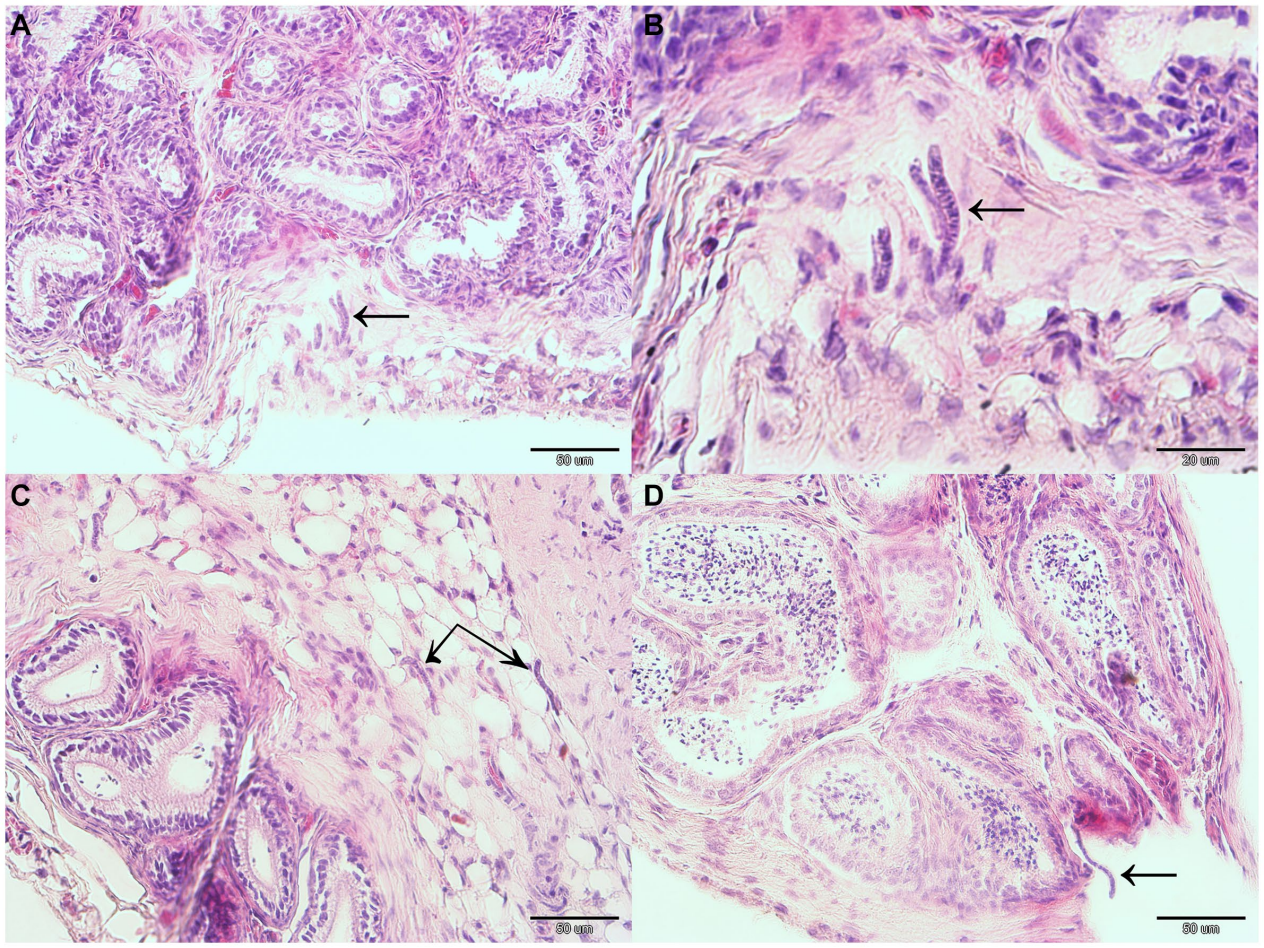
Microfilariae located within reproductive organ tissues of a male parti-coloured bat (*Vespertilio murinus*). Microfilariae (indicated with black arrows) can be seen in the testicular connective tissue **(A,B)** and epidydimal connective and fat tissue **(C,D)**.

**Figure 3:**
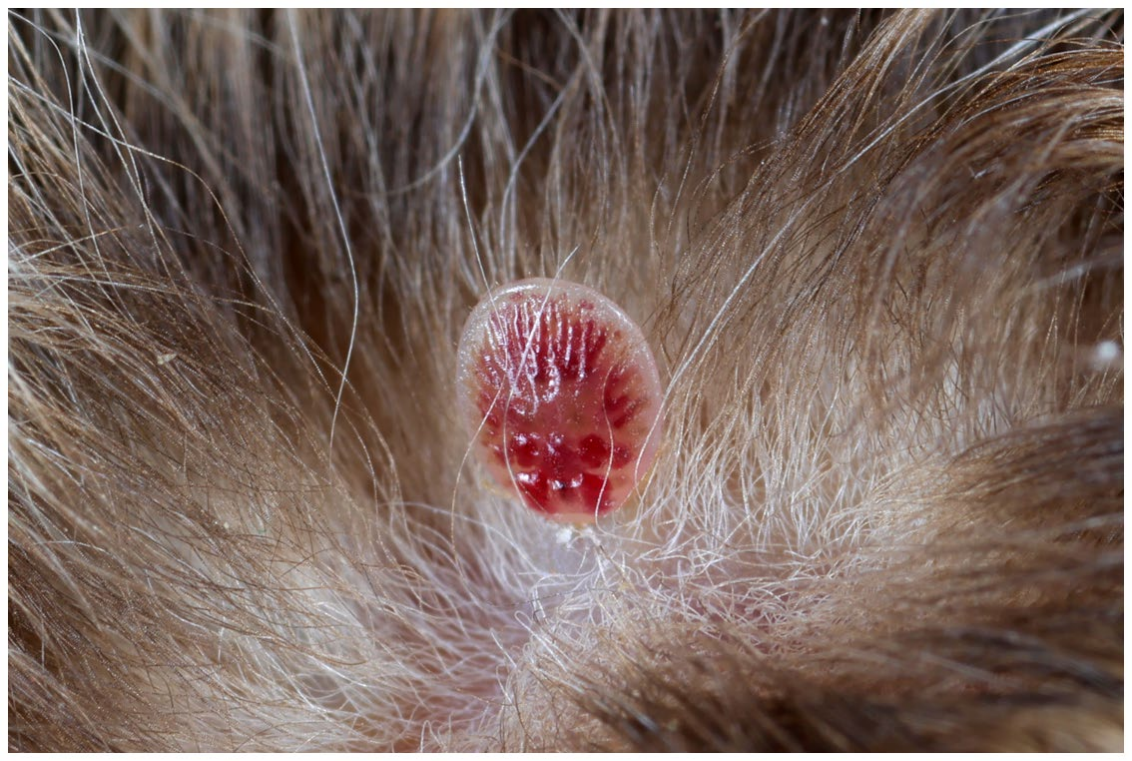
A mildly engorged argasid mite larva attached to the skin of a male parti-coloured bat (*Vespertilio murinus*).

Gross pathology examination revealed haemorrhagic gastroenteritis and bloating. A total of eight adult filarial worms were found in the peritoneal cavity, close to the stomach (Figure 1B). Microscopic examination revealed microfilariae in the testicular connective tissue (Figures 2A,B) and epidydimal connective and fat tissues (Figures 2C,D), with no inflammatory response. However, microfilariae were not observed in histopathological sections of the lungs, liver and kidneys, or the blood vessels within these organs. Three argasid mite larvae, mildly engorged with blood, were found attached to the skin of the bat (Figure 3).

### 3.3. Identification of intestinal microbial agents

**Figure 4:**
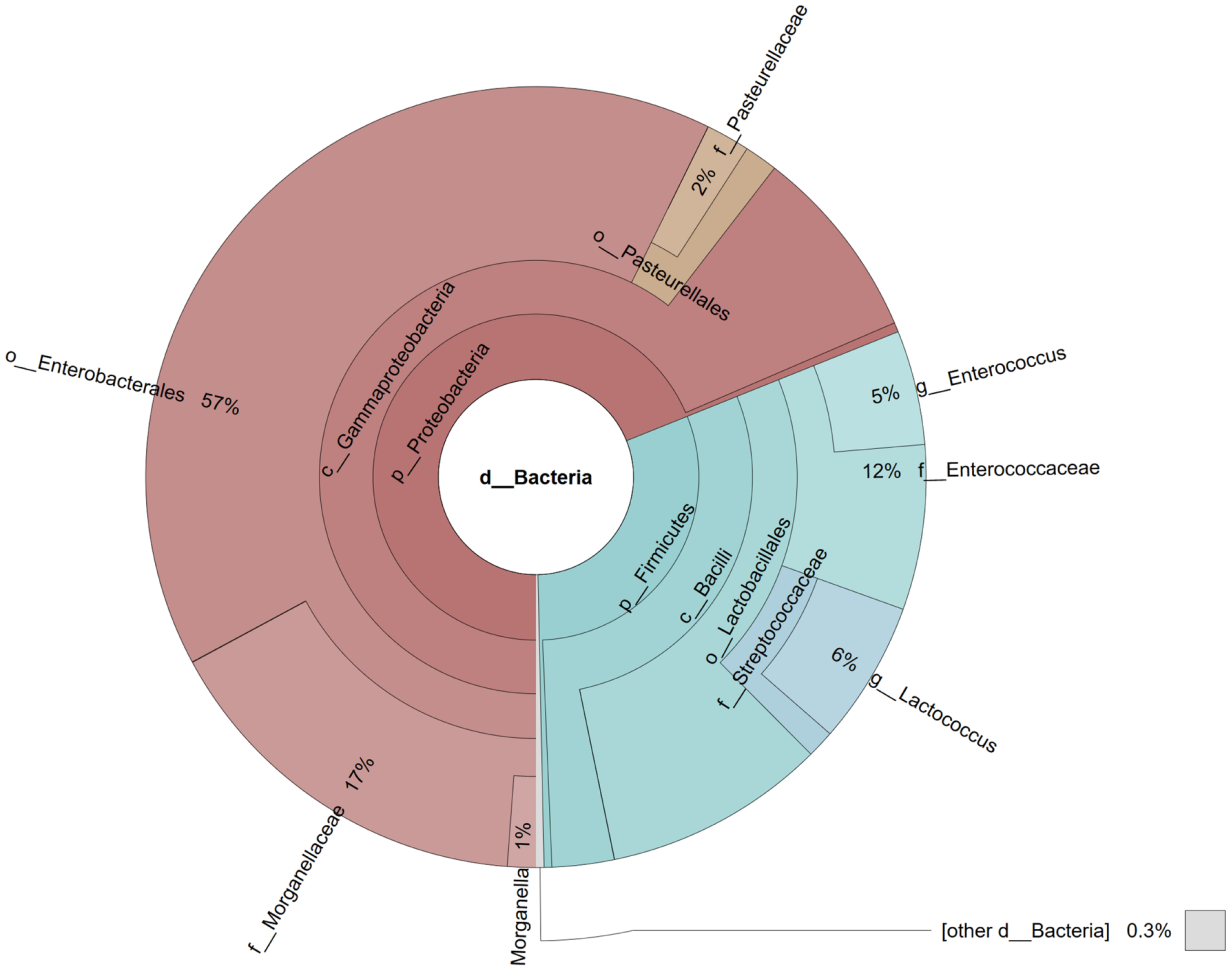
Pie chart illustrating the gut microbial community of a male parti-coloured bat (*Vespertilio murinus*) that died of dysmicrobia and haemorrhagic gastroenteritis.

A total of 87,368 reads were obtained after sequencing the library. During the bioinformatic analysis, singletons were filtered away and the remaining reads assigned to 17 operational taxonomic units (OTUs). At the phylum level, Proteobacteria were the most abundant taxon (68.94%), followed by Firmicutes (30.73%), while at the class level, most reads were assigned to *Gammaproteobacteria* (68.54%) and *Bacilli* (30.41%; Figure 4). Most *Gammaproteobacteria* belonged to the orders *Enterobacterales* (57.28%) and *Pasteurellales* (3.19%), while the majority of *Bacilli* were represented by Lactobacillales (27.84%), with 17.13% identified as members of the Morganellaceae family with *Morganella* (1.17%) the dominating genus, 11.57% assigned to the *Enterococcaceae* family with the *Enterococcus* genus (4.74%) predominant, 7.06% assigned to *Streptococcaceae* (5.92% *Lactococcus*), and 1.82% assigned to the *Pasteurellaceae* family (Figure 4).

### 3.4. Molecular identification of the filarial parasite and screening for *Wolbachia*

The PCR assay targeting COI in DNA extracted from adult worms produced two high quality sequences, while molecular clones of COI from the bat’s testicular microfilariae produced eight high quality sequences. All sequences were deposited into the GenBank database under accession numbers OP796363 to OP796372. These sequences showed 91.08 to 91.38% genetic identity with the *Litomosa* sp. WB-2018 COI gene (sequence ID MH411205) from worms detected as filarial infection in the thoracic cavity of a Bulgarian Savi’s pipistrelle bat (*Hypsugo savii*). The argasid mite larvae proved negative for filarial DNA, while the adult filarial worms from the bat’s peritoneal cavity tested negative for presence of *Wolbachia.*

## 4. Discussion

### 4.1. Bat semen quality

To the best of our knowledge, this is the first report on semen quality for *V. murinus*. As we lack deeper knowledge about semen quality in most chiropteran species, and no study evaluating sperm quality of vespertilionid bats has ever been published, our data cannot be directly compared. However, the semen concentration and sperm motility values measured in our *V. murinus* male appear high compared with phyllostomid (23, 29, 30), vespertilionid and rhinolophid (31) and pteropid (32) bats.

Spermatozoa and seminal fluid are both standard parts of ejaculated semen, with the seminal fluid acting as a complex medium for the transport, protection and nourishment of the spermatozoa. However, the seminal fluid can also provide the same functions for semen-borne pathogens (33). For example, the seminal fluid of mammals is rich in fructose, the major source of energy metabolism for spermatozoa (34). In addition to glucose, however, filarial parasites are also able to utilise fructose as an energy source (35), suggesting that bat seminal fluid may facilitate filarial parasite transmission. Likewise, bat uterine fluids are high in fructose of both male and female contribution (36, 37), and these may contribute to the ability of hibernating female bats to store sperm for months as part of their delayed fertilisation reproduction strategy (38). We may hypothesise, therefore, that each mating in bats showing these reproductive physiology traits allows microfilariae to enter the uterus, where they can first infect the female and later, the developing embryo and/or foetus. Importantly, the sperm storage organs in male and female bats, i.e., the epididymis and uterus, are both immune-privileged sites (37, 39) in which both germ cells and the parasites may be protected from immune attack.

### 4.2. Pathological examination

Based on gross pathology, the primary cause of the bat’s death was associated with haemorrhagic gastroenteritis and bloating. Outbreaks of haemorrhagic diarrhoea in groups of captive handicapped bats have been recognised in association with bacterial infection, with contaminated mealworms as a likely source of infection (22). Presence of adult filarial worms in the peritoneal cavity, however, induced no signs of disease, while the microfilariae observed in the testicular tissue during histopathology were not associated with any signs of inflammation, suggesting that immunological tolerance may be limiting the harm caused by the parasite (40). Unfortunately, there is limited information available on pathogenicity of filarial infections in bats. However, Rendón-Franco et al. (12) noticed signs of weakness, tachypnoea and patagial haemorrhages in two male Aztec fruit-eating bats (*Artibeus aztecus*) captured in Mexico, contrary to our findings. Histopathology of organs collected from these bats revealed lesions such as multifocal exudative pneumonia, neutrophilic inflammatory infiltration and oedema of the lungs associated with *Litomosoides* sp. microfilaraemia.

In vespertilionid bats, the testes descend into the scrotum and are covered by the tunica vaginalis, a pigmented sheet of peritoneum (41). During foetal development, the testis descend through the respective inguinal canals through an extension of the peritoneal cavity into the scrotum called the processus vaginalis. We hypothesise that microfilariae are able to invade the male bat’s testes by migrating from the site of release by adult females in the peritoneal cavity along this peritoneal tunnel route. An alternative migration route used by microfilariae to arrive at the processus vaginalis may be *via* the lymphatic vessels; observed, for example, in *Brugia malayi* infections (42). Interestingly, microfilariae of *Wuchereria bancrofti* have also been shown to infect testicular tissue (43, 44) and follicular fluid in humans (45).

### 4.3. Identification of intestinal microbial agents

The gut microbial community structure of insectivorous bats is highly specific, differing clearly from the microbiome of other mammalian species. Regarding the bacterial phylum, Proteobacteria and Firmicutes are most abundant, with the bacterial families *Enterobacteriaceae*, *Enterococcaceae*, *Lactobacillaceae* and *Fusobacteriaceae* dominating (46). Microbiome analysis of the *V. murinus* in this study, which was suffering from haemorrhagic gastroenteritis, indicated a low diversity of bacterial taxa comprising just two classes, three orders and five families. The dominant family in this case were the Morganellaceae, mainly comprising the genus *Morganella*, which is normally a rather sporadic taxon in the chiropteran gut microbiome (47) and may have been the causative agent of the haemorrhagic gastroenteritis (48). A decrease in intestinal microbiome diversity is often associated with a state of dysbiosis. In humans, for example, an intestinal microbiome composition similar to that observed in our *V. murinus* bat has been referred to as ‘low diversity dysbiosis’ (49). Dysmicrobia, and subsequent haemorrhagic gastroenteritis, could result from stress, a significant problem for wild animals kept in captivity (50). Unbalanced and/or highly nutrient-rich diet could be another reason for dysmicrobia (51).

### 4.4. Molecular identification of the filarial parasite

A BLAST search of the parasites collected in the peritoneal cavity of our *V. murinus* generated results showing 91% genetic identity with a filarial parasite *Litomosa* sp. described in *H. savii*. While sequence divergencies of COI readily discriminate closely related species (52), there are unfortunately no COI sequences available in the GenBank database for *L. ottavianii*, *L. vaucheri* known from *V. murinus* and *Litomosa* sp. from *V. sinensis* (9, 10, 20). In fact, sequence of *Litomosa* sp. from *Hypsugo savii* is the only available COI sequence of genera *Litomosa* and *Litomosoides* obtained from European or Asian bats. While a PCR assay targeting COI confirmed that the adult worms from the bat’s peritoneal cavity and testicular microfilariae were of the same filarial species, morphological characteristics of the worms from our *V. murinus* bat differed from those described previously (9, 10, 20), suggesting that the parasite is a novel filarial species (a full description of this new species will be included in a separate manuscript), together with the parasite’s molecular phylogeny (Pikula et al., in preparation).

### 4.5. Screening the filarial parasite for *Wolbachia*

The adult filarial worms in the present study were all free of the endosymbiont *Wolbachia*, as were two bat filarial species (i.e., *Litomosoides yutajensis* and *Litomosa chiropterorum*) previously screened for *Wolbachia* (5, 53). It would appear, therefore, that presence of *Wolbachia* endosymbionts is not essential for filarial physiology (5). Considering the strict vertical transmission of endosymbiotic *Wolbachia* between females and their offspring, it would be expected that the phylogenies of filarial hosts and their symbionts match (27); however, phylogenetic analyses have indicated that some filarial lineages may have lost *Wolbachia* during their evolution (54, 55). Interestingly, *Wolbachia*-like DNA sequences have been found within the genomes of some *Wolbachia*-free filarial nematodes, suggesting ancient infection with the endosymbiont and horizontal genetic transfer, potentially explaining how these nematodes support their physiological needs (56).

### 4.6. The biology and life cycle of filarial nematodes

Microfilariae released by females at the site of infection spread within the host *via* blood and lymphatic circulation (57). While filarial infections are generally known to be vectored by different arthropods, just one macronyssid ectoparasite, the tropical rat mite (*Ornithonyssus bacoti*) has been experimentally confirmed as playing a role in the larval biology of a *Litomosoides* filaria of Costa Rican common fruit bats (*Artibeus jamaicensis*) (58). Vectors feeding on the host ingest microfilariae which then develop into infective L3 larvae. These larvae may then enter and infect a new host when the vector feeds on another bat. Further steps in the life cycle include migration of larvae to a specific site in the body, maturation, mating and production of microfilariae by adult females. Sometimes, microfilariae may be present in blood; however, no adult worms have been discovered during bat necropsy examinations, suggesting longer survival of microfilariae than adults in the host (58).

Argasid mite larvae attached to the *V. murinus* male in this study proved negative for filarial DNA; however, it is not known whether these ectoparasites serve as vectors for *Litomosa* microfilariae. Considering the host–parasite system dependent on a vector, it is possible that the social structure and roosting behaviour of bats could reduce opportunities for ectoparasite transmission (59) and infection with vector-borne filarial larvae because in *V. murinus* the males separate from females and move to different habitats/sites for most of the year, where they remain territorial and roost individually throughout the autumn mating period (21).

Temperate vespertilionid bats are monoestrous and mating tends to be polygynous and promiscuous (37). Hypothetically, sexual transmission may allow vector-borne filarial parasites to cycle in conditions where there is a reduced chance of reaching new hosts *via* transmission through ectoparasites. Indeed, intensity of transmission through this modified route may even be promoted by the bat’s polygynous mating system, in which the infected male mates with many females during the autumn swarming. It remains unclear whether microfilariae sexually transmitted to females then continue their development. Most probably not, as microfilariae can continue further development only following ingestion by a proper vector (58). Even if they could not continue their development without the invertebrate vector, microfilariae may be able to get into the bloodstream of the female bat and continue the classic life cycle of filarial nematodes because microfilariae infecting females in large maternity colonies may have increased opportunities for ingestion by a proper vector, allowing it to develop further and enter a new host to mature in. This would be similar to the situation whereby canine puppies suffer transplacental infection with microfilariae of the heartworm *Dirofilaria immitis* (60). Likewise, microfilariae of *Wuchereria bancrofti* may cross the human placenta. As a result of *in utero* exposure, children born to infected mothers may be tolerant and more likely to become infected later in their life (61).

## 5. Conclusion

In the present case report, we provide the first description of semen quality of a parti-coloured bat and evidence for (a) adult filarial nematodes, genetically identified as *Litomosa* sp., residing in the peritoneal cavity of an adult male *V. murinus* bat, and (b) microfilariae of the filarial nematode within testicular tissues and in the bat’s semen, suggesting that this nematode may also be a semen-borne pathogen. While comparative data is lacking, presence of microfilariae may be expected to decrease semen quality and transmission *via* this route may challenge the success of reproductive events in females after mating. Further investigation will be necessary to better understand this filarial species life cycle and gender differences in disease prevalence.

## Data availability statement

The original contributions presented in the study are publicly available. This data can be found at: https://www.ncbi.nlm.nih.gov/sra; SRR25944610.

## Ethics statement

The animal study was approved by Ethical Committee of the University of Veterinary Sciences Brno, Czechia. The study was conducted in accordance with the local legislation and institutional requirements.

## Author contributions

JP: Conceptualization, Data curation, Formal analysis, Funding acquisition, Investigation, Project administration, Supervision, Visualization, Writing – original draft, Writing – review & editing. VP: Investigation, Methodology, Visualization, Writing – review & editing. HB: Formal analysis, Investigation, Writing – review & editing. MB: Investigation, Writing – review & editing. SB: Data curation, Formal analysis, Investigation, Writing – review & editing. RB: Investigation, Writing – review & editing. OD: Investigation, Methodology, Writing – review & editing. PJ: Methodology, Writing – review & editing. SM: Investigation, Writing – review & editing. MN: Investigation, Writing – review & editing. VS: Investigation, Writing – review & editing. KZ: Funding acquisition, Investigation, Writing – review & editing. JZ: Investigation, Writing – review & editing.

## Funding

The author(s) declare financial support was received for the research, authorship, and/or publication of this article. This research was supported through Project IGA 216/2021/FVHE and the Czech Science Foundation (grant no. 21-12719S). The funder had no role in the study design, data analysis, the decision to publish or the preparation of the manuscript.

## Conflict of interest

The authors declare that the research was conducted in the absence of any commercial or financial relationships that could be construed as a potential conflict of interest.

## Publisher’s note

All claims expressed in this article are solely those of the authors and do not necessarily represent those of their affiliated organizations, or those of the publisher, the editors and the reviewers. Any product that may be evaluated in this article, or claim that may be made by its manufacturer, is not guaranteed or endorsed by the publisher.
